# Toxicity of external beam accelerated partial-breast irradiation (APBI) in adjuvant therapy of early-stage breast cancer: prospective randomized study

**DOI:** 10.1186/s13014-024-02412-x

**Published:** 2024-02-03

**Authors:** Petr Burkon, Iveta Selingerova, Marek Slavik, Milos Holanek, Miroslav Vrzal, Oldrich Coufal, Katerina Polachova, Petr Muller, Pavel Slampa, Tomas Kazda

**Affiliations:** 1 Department of Radiation Oncology, Masaryk Memorial Cancer Institute, Brno, Czech Republic; 2 Department of Radiation Oncology, Faculty of Medicine, Masaryk University, Brno, Czech Republic; 3 Research Centre for Applied Molecular Oncology (RECAMO), Masaryk Memorial Cancer Institute, Zluty kopec 7, 656 53 Brno, Czech Republic; 4 Department of Mathematics and Statistics, Faculty of Science, Masaryk University, Brno, Czech Republic; 5 Department of Comprehensive Cancer Care, Masaryk Memorial Cancer Institute, Brno, Czech Republic; 6 Department of Comprehensive Cancer Care, Faculty of Medicine, Masaryk University, Brno, Czech Republic; 7 Department of Surgical Oncology, Masaryk Memorial Cancer Institute, Brno, Czech Republic; 8 Department of Surgical Oncology, Faculty of Medicine, Masaryk University, Brno, Czech Republic

**Keywords:** Early breast cancer, Adjuvant radiotherapy, Surgical bed, APBI, External beam radiotherapy

## Abstract

**Background:**

Accelerated partial breast irradiation (APBI) is an alternative breast-conserving therapy approach where radiation is delivered in less time compared to whole breast irradiation (WBI), resulting in improved patient convenience, less toxicity, and cost savings. This prospective randomized study compares the external beam APBI with commonly used moderate hypofractionated WBI in terms of feasibility, safety, tolerance, and cosmetic effects.

**Methods:**

Early breast cancer patients after partial mastectomy were equally randomized into two arms– external APBI and moderate hypofractionated WBI. External beam technique using available technical innovations commonly used in targeted hypofractionated radiotherapy to minimize irradiated volumes was used (cone beam computed tomography navigation to clips in the tumor bed, deep inspiration breath hold technique, volumetric modulated arc therapy dose application, using flattening filter free beams and the six degrees of freedom robotic treatment couch). Cosmetics results and toxicity were evaluated using questionnaires, CTCAE criteria, and photo documentation.

**Results:**

The analysis of 84 patients with a median age of 64 years showed significantly fewer acute adverse events in the APBI arm regarding skin reactions, local and general symptoms during a median follow-up of 37 months (range 21–45 months). A significant difference in favor of the APBI arm in grade ≥ 2 late skin toxicity was observed (*p* = 0.026). Late toxicity in the breast area (deformation, edema, fibrosis, and pain), affecting the quality of life and cosmetic effect, occurred in 61% and 17% of patients in WBI and APBI arms, respectively. The cosmetic effect was more favorable in the APBI arm, especially 6 to 12 months after the radiotherapy.

**Conclusion:**

External APBI demonstrated better feasibility and less toxicity than the standard regimen in the adjuvant setting for treating early breast cancer patients. The presented study confirmed the level of evidence for establishing the external APBI in daily clinical practice.

**Trial registration:**

NCT06007118.

**Supplementary Information:**

The online version contains supplementary material available at 10.1186/s13014-024-02412-x.

## Background

The preferred treatment option for most patients with early breast cancer (BC) is breast-conserving therapy (BCT) [1], consisting of partial mastectomy and subsequent breast irradiation. Conventionally fractionated whole-breast irradiation (WBI) with or without an additional tumor bed dose, time burdening patients, usually taking 5–7 weeks, was standard for decades. Large phase III trials have proved that overall irradiation time could be reduced using hypofractionated WBI without compromising local control and warranting a good safety profile [2, 3].

Partial-breast irradiation (PBI) has been introduced as an alternative approach for adjuvant radiotherapy (RT) after partial mastectomy in selected low-risk early BC patients. Compared with WBI, estimated advantages of PBI include shorter overall treatment time when RT is accelerated (APBI, accelerated partial breast irradiation), improved safety profile, and potential cost reduction [4]. Several large phase III trials demonstrated the noninferiority of PBI versus WBI in terms of local recurrence (LR) and similar or reduced toxicity at five years [5–9]. Recommendations for APBI have been published by both the American Society for Radiation Oncology and the Groupe Européen de Curiethérapie-European Society for Radiotherapy Oncology [10–12].

There are several technical possibilities for APBI, such as interstitial or intracavitary brachytherapy (BRT) or external beam irradiation. Three-dimensional conformal radiotherapy (3D-CRT) has been associated with a higher risk of skin reactions and worse cosmetic results [13–15], particularly because of the need to accommodate extra safety margins to compensate for inaccuracies during irradiation. APBI, based on the principles of hypofractionation, has the advantages of being less invasive and faster due to focusing on the target, using a higher radiation dose per fraction, and reducing the dose to the surrounding normal tissues. Moreover, it improves the accuracy of treatment through the different currently available machine devices, such as image-guided radiotherapy (IGRT) aimed at clips in the tumor bed using CT devices on the platform of linear accelerator (cone beam computed tomography, CBCT); [16] irradiation in deep inspiration breath hold (DIBH technique) [17, 18] to stop breast movements during breathing; fast and accurate dose application using arc therapy (volumetric modulated arc therapy, VMAT) [19]; radiation beams without a homogenizing filter with a high dose rate (flattening filter free beams, FFF) [20] or correction of the patient’s position with six degrees of freedom robotic treatment couch (6DoF couch) [21].

The aim of this prospective randomized single-institution study conducted by the Department of Radiation Oncology at Masaryk Memorial Cancer Institute (MMCI) in Brno, Czechia, was to compare APBI (5 fractions) of the early BC patients with the currently more commonly used moderate hypofractionated WBI regimen (20 fractions) [22]. The main objective was to evaluate the feasibility, safety, tolerance, and cosmetic effects of APBI and, thus, to increase the evidence for establishing this technique in indicated patients into daily clinical practice.

## Methods

### Patients and study design

Patients with early-stage BC referred for adjuvant RT were randomly assigned to the following two treatment arms: APBI arm (irradiation of tumor bed, 30 Gy in 5 fractions, referred as study arm) or WBI arm (moderate hypofractionated irradiation of the whole breast with a boost to the tumor bed, 40 Gy in 15 fractions followed by 10 Gy in 5 fractions). Written informed consent was received from each patient prior to enrolment. The study was approved by the Ethical Board of Masaryk Memorial Cancer Institute (MMCI; approval No. 2017/1889/MOU) and registered at ClinicalTrials.gov (NCT06007118).

The inclusion criteria were: age ≥ 50 years; Karnofsky index > 70; breast-conserving surgery; DCIS G1/2 ≤ 25 mm with negative margins (≥ 3 mm) or invasive (non-lobular) luminal-like HER2 negative carcinoma ≤ 20 mm with negative margins (≥ 2 mm) without LVI; performing of axillary dissection (≥ 6 negative lymph nodes) or negative sentinel node biopsy. The exclusion criteria were: prior chest or breast surgery; absence of surgical clips in tumor bed; multifocal or multicentric involvement; factors contraindicating RT; known *BRCA1/2* or other mutation in high penetrating genes; neoadjuvant therapy; prior RT; adjuvant chemotherapy.

### Randomization

A stratified permuted block randomization scheme with a block size of four was used to assign patients to arms in a 1:1 ratio. The treatment group assignment was not blinded. Stratification factors were (1) surgery bed size measured by the longest distance of surgery clips placed for radiotherapy navigation (< 30 mm or ≥ 30 mm), and (2) phototype (light (phototype I/II) or dark (phototype III/IV)).

### Treatment

Safety and accuracy of treatment were achieved by ensuring reliable and reproducible immobilization (frameless fixation with Orfit Industries and CIVCO Medical Solutions vacuum-formable mattresses) and using all technical machine devices described above (cone beam computed tomography navigation to clips in the tumor bed, deep inspiration breath hold technique, volumetric modulated arc therapy dose application, using flattening filter free beams and the six degrees of freedom robotic treatment couch). CT scans with 2 mm thick slices, including the curve of respiratory movements, were sent to the radiotherapy planning system. For WBI, the clinical target volume (CTV) was defined by the residual parenchyma of the gland. The planning target volume (PTV) was created by expanding CTV by 10 mm in all directions. For both arms, tumor bed CTV encompasses the excision cavity with a 10 mm margin. Visible cavity and clips placed to the cavity borders during surgery were used to define the CTV. The CTV did not include the chest wall and pectoralis muscles and was limited to 5 mm from the skin surface. In the APBI arm, PTV was delineated with a 3-mm extent in all directions from CTV to accommodate possible set-up errors. The PTV was also limited to 5 mm from the skin surface. The prescribed radiation dose was planned for this final PTV.

The WBI arm patients were irradiated with a moderate hypofractionated mode within 20 working days [3]. The whole breast was irradiated with 40.05 Gy in 15 fractions, followed by a boost to the tumor bed with 10 Gy in 5 fractions. The APBI arm patients received a total dose of 30 Gy administered in 5 fractions over five consecutive days, allowing for a potential break over the weekend, provided that there were at least two consecutive fractions before or after the weekend break. The dose distribution and beam arrangement of APBI are presented in Supplementary Fig. 1. Treatment plans were created using Eclipse planning system version 15.6 (Varian Medical Systems, Palo Alto CA) using the AAA algorithm. VMAT technique (2–3 partial arcs) and a high dose rate 6 MV beam without a homogenization filter (FFF) were used [19, 20, 23]. Adequate target coverage was achieved when the prescribed dose covered 95% of the PTV. A dose gradient was also assessed, and the treatment plans should meet the number of organs at risk dose-volume constraints [24–27] based on published studies (Supplementary Table 1). The treatment was delivered by the linear accelerator Varian TrueBeam STX v. 2.5.(Varian Medical Systems, Palo Alto CA).

### Follow-up and outcomes

Evaluation and study assessments were scheduled prior to RT (B, baseline), at the end of RT (M0), and in 1 (M1), 3 (M3), 6 (M6), 9 (M9), 12 (M12) months after RT, in the second year every four months and then every six months.

The primary endpoint of the study was toxicity evaluated by CTCAE (Common Terminology Criteria for Adverse Events). Acute toxicity was defined as adverse reactions occurring within three months after RT, and late toxicity occurs during the next follow-up. The grades presented are the patient’s worst toxicity at any time point. Secondary endpoints were quality of life (Qol) measured with the official Czech translation of EORTC QoL questionnaires [28–30] (EORTC QLQ-C30), including a special module for patients with BC (Breast QLQ-BR45); cosmetic effect independently evaluated by patient, physician, and nurse scored using Harvard scale (4-point Likert scale) [31]; change in breast appearance (photographic) assessed on a 3-point graded scale (none, mild, marked); and economy burden of patients evaluated at the end of RT by a 4-point graded scale (none, mild, middle, significant). For future evaluation of subsequent endpoints (recurrence-free survival, disease-specific survival, ipsilateral breast-recurrence rate, distant disease-free interval, and overall survival), patients will be followed up according to standards of care. Six months after RT, pulmonary toxicity was assessed based on clinical examination and computed tomography (CT) scan in the treatment position.

### Sample size and statistical analysis

The study was designed to assess the noninferiority of APBI to relative WBI in terms of the grade ≥ 2 late skin toxicity involving events between 6 months and two years after RT. Assuming an incidence of late skin toxicity in the WBI arm of 15% and an expected incidence in APBI of 5%, and based on two proportion z-test with a noninferiority margin of 10%, one-sided significance level of 5%, test power of 90%, and 10% dropout rate, 84 patients (42 in each arm) were required.

Patient and treatment characteristics were described using the standard summary statistics, i.e., median and interquartile range (IQR) for continuous variables and numbers and percentages for categorical variables. Depending on the nature of the data, Fisher’s exact or chi-square test for categorical variables and nonparametric Mann-Whitney test for continuous variables were used to compare arms. A significance level of 5% was considered for all statistical tests, and R statistical software version 4.3.1 [32] was used.

## Results

**Fig. 1:**
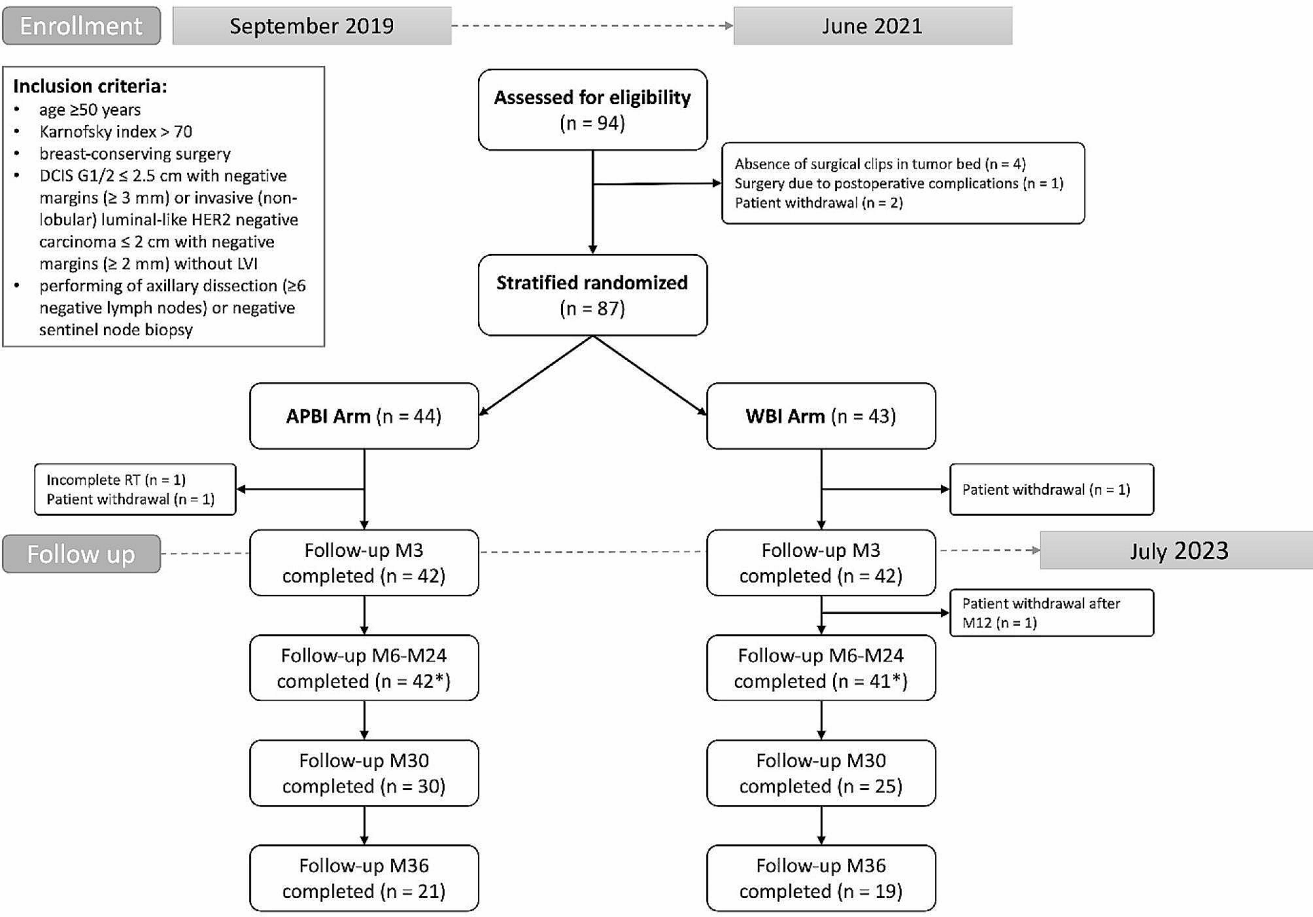
Consort Diagram. *Some patients skipped one visit (APBI Arm– one patient M9 and one patient M20, WBI Arm– one patient M6 and one patient M12). Abbreviations: APBI = accelerated partial breast irradiation, WBI = whole breast irradiation, DCIS = Ductal Carcinoma In Situ

From September 2019 to June 2021, 87 patients were enrolled and randomized. Of these, three were excluded from the final evaluation due to insufficient follow-up (Fig. 1). In the analyzed cohort, 42 patients were assigned to the APBI arm and 42 to the WBI arm. Median follow-up was 37 months (range 21–45 months). No recurrence, regional or distant relapse of the disease was detected in all patients during follow-up.

Baseline patients’ characteristics are summarized in Table 1. No significant difference was observed between arms. The majority of enrolled patients had NST tumors (79%), up to 10 mm (50%), of nuclear grade G1 (59%), with low Ki67 status (55%). The median negative resection margin was 5 and 5.5 mm in the APBI and WBI groups, respectively. 74% of patients had concurrent endocrine therapy.

**Table 1:** Baseline characteristics by study arm

		APBI *N* = 42	WBI *N* = 42	*p*-value
**Age (years)**	Median (Range)	65 (52, 77)	64 (51, 81)	0.070
**Phototype**	I/II	19 (45%)	20 (48%)	0.827
	III/IV	23 (55%)	22 (52%)	
**Laterality**	left	18 (43%)	21 (50%)	0.512
	right	24 (57%)	21 (50%)	
**Grade**	G1	25 (60%)	24 (57%)	0.825
	G2	17 (40%)	18 (43%)	
**Histology**	DCIS	2 (4.8%)	2 (4.8%)	0.911
	NST	34 (81%)	32 (76%)	
	Other	6 (14%)	8 (19%)	
**Tumor size (mm)**	0–9	24 (57%)	18 (43%)	0.422
	10–14	13 (31%)	17 (40%)	
	15–20	5 (12%)	7 (17%)	
**ER (%)**	90	0 (0%)	3 (7.1%)	0.121
	95	2 (4.8%)	4 (9.5%)	
	100	40 (95%)	35 (83%)	
**PR (%)**	< 10	8 (19%)	9 (21%)	0.267
	10–79	10 (24%)	16 (38%)	
	80–100	24 (57%)	17 (40%)	
**Ki67 (%)**	< 15	21 (50%)	25 (60%)	0.583
	15–20	13 (31%)	12 (29%)	
	> 20	8 (19%)	5 (12%)	
**Number of sentinel lymph nodes removed**	1	25 (60%)	22 (55%)	0.462
	2	8 (19%)	12 (30%)	
	> 2	9 (21%)	6 (15%)	
	Omitted	0	2	
**Negative resection margin (mm)**	Median (Range)	5 (2, 10)	5.5 (2, 15)	0.718
**Cavity size (mm)**	Median (Range)	30 (16, 69)	31 (13, 65)	0.629
	< 30	22 (52%)	21 (50%)	0.827
	≥ 30	20 (48%)	21 (50%)	
**Endocrine therapy during RT**	None	9 (21%)	4 (9.5%)	0.345
	AI	4 (9.5%)	5 (12%)	
	Tamoxifen	29 (69%)	33 (79%)	

Abbreviations: APBI = accelerated partial breast irradiation, WBI = whole breast irradiation, NST = non-specified tumor histology, ER = estrogen receptor, PR = progesterone receptor, RT = radiotherapy, AI = aromatase inhibitor, DCIS = Ductal Carcinoma In Situ

Baseline dose-volume characteristics are summarized in Table 2. The CTV volume median in the study arm was 58.5 cc (range 25.7–141.0), and the PTV volume median was 86.5 cc (range 40.9–189.9). In the control group, the median size of the CTV boost was 14.5 cc (range 4.2–59.4), and PTV boost was 69.0 cc (range 33.9–167.7).

**Table 2:** Dose-volume characteristics

Median (range)	APBI *N* = 42	WBI *N* = 42
**Breast volume (cc)**	858 (418, 1,577)	912 (269, 2,371)
**CTV (cc)**	58.5 (25.7, 141.0)	912.2 (269.3, 2,370.8)
**CTV boost (cc)**		14.5 (4.2, 59.4)
**CTV D**_**min**_ **(Gy)**	29.3 (28.0, 30.2)	48.5 (32.5, 50.7)
**CTV D**_**near−min**_ **(Gy)**	30.2 (29.9, 31.0)	49.0 (44.4, 51.1)
**PTV (cc)**	86.5 (40.9, 189.9)	1,251.8 (496.2, 2,753.1)
**PTV boost (cc)**		69.0 (33.9, 167.7)
**PTV D**_**min**_ **(Gy)**	26.7 (23.2, 28.3)	45.9 (18.1, 49.2)
**PTV D**_**near−min**_ **(Gy)**	29.6 (29.2, 29.8)	48.2 (42.1, 50.1)
**D**_**max**_ **(Gy)**	32.1 (31.5, 32.7)	52.1 (42.0, 53.9)

Abbreviations: APBI = accelerated partial breast irradiation, WBI = whole breast irradiation, Gy = Gray, CTV = clinical target volume, PTV = planning target volume, D_min_ = minimum dose of the volume, D_near−min_ = near-minimum dose of the volume, D_max_ = maximum dose of the volume. D_near−min_ referred according to ICRU report 83

The radiation toxicities at acute and late periods are presented in Table 3. Significantly fewer acute adverse events were observed in the APBI arm regarding skin reactions and local and general symptoms (*p* < 0.001 for all). Skin side effects of grade ≥ 2 were significantly less often in the APBI arm in terms of erythema (*p* < 0.001), hyperpigmentation (*p* = 0.002), and desquamation (*p* = 0.012).

**Table 3:** Acute and late toxicities by study arm

	APBI, *N* = 42			WBI, *N* = 42			*p*-value	
	Grade 1	Grade 2	Grade 3	Grade 1	Grade 2	Grade 3	Any grade	Grade ≥ 2
**Acute period**								
**General**	9 (21%)	—	—	23 (55%)	3 (7.1%)	—	**< 0.001**	0.241
Anorexia/dyspepsia	1 (2.4%)	—	—	—	—	—	—	—
Fatigue	8 (19%)	—	—	22 (52%)	3 (7.1%)	—	**< 0.001**	0.241
Flu like symptoms	1 (2.4%)	—	—	—	—	—	—	—
Gastrointestinal pain	—	—	—	1 (2.4%)	—	—	—	—
Nausea	—	—	—	1 (2.4%)	—	—	—	—
Weight loss	—	—	—	1 (2.4%)	—	—	—	—
**Local (breast area)**	16 (38%)	—	—	31 (74%)	4 (9.5%)	—	**< 0.001**	0.116
Deformation	2 (4.8%)	—	—	8 (19%)	1 (2.4%)	—	**0.048**	—
Edema	3 (7.1%)	—	—	15 (36%)	2 (4.8%)	—	**< 0.001**	0.494
Pain	5 (12%)	—	—	23 (55%)	2 (4.8%)	—	**< 0.001**	0.494
Tumor bed fibrosis	8 (19%)	—	—	12 (29%)	1 (2.4%)	—	0.314	—
**Regional**	3 (7.1%)	—	—	9 (21%)	—	—	0.116	—
Cough	2 (4.8%)	—	—	4 (9.5%)	—	—	0.676	—
Dyspnea	2 (4.8%)	—	—	2 (4.8%)	—	—	> 0.999	—
Chest wall pain	1 (2.4%)	—	—	3 (7.1%)	—	—	0.616	—
Palpitations/cardiac pain	—	—	—	1 (2.4%)	—	—	—	—
Pneumonitis	—	—	—	1 (2.4%)	—	—	—	—
Upper limb lymphedema	—	—	—	1 (2.4%)	—	—	—	—
**Skin**	29 (69%)	—	—	19 (45%)	22 (52%)	1 (2.4%)	**< 0.001**	**< 0.001**
Desquamation	—	—	—	14 (33%)	7 (17%)	—	**< 0.001**	**0.012**
Dryness	2 (4.8%)	—	—	30 (71%)	1 (2.4%)	—	**< 0.001**	—
Edema	5 (12%)	—	—	20 (48%)	2 (4.8%)	—	**< 0.001**	0.494
Erythema	21 (50%)	—	—	24 (57%)	17 (40%)	1 (2.4%)	**< 0.001**	**< 0.001**
Hyperpigmentation	11 (26%)	—	—	29 (69%)	9 (21%)	—	**< 0.001**	**0.002**
Tenderness	6 (14%)	—	—	33 (79%)	1 (2.4%)	—	**< 0.001**	—
**Late period**								
**General**	5 (12%)	—	—	3 (7.3%)	—	—	0.713	—
Fatigue	5 (12%)	—	—	3 (7.3%)	—	—	0.713	—
**Local (breast area)**	7 (17%)	—	—	25 (61%)	3 (7.3%)	—	**< 0.001**	0.116
Deformation	—	—	—	10 (24%)	2 (4.9%)	—	**< 0.001**	0.241
Edema	1 (2.4%)	—	—	7 (17%)	2 (4.9%)	—	**0.007**	0.241
Pain	3 (7.1%)	—	—	15 (37%)	—	—	**0.001**	—
Tumor bed fibrosis	4 (9.5%)	—	—	11 (27%)	1 (2.4%)	—	**0.028**	0.494
**Regional**	3 (7.1%)	—	—	3 (7.3%)	—	—	> 0.999	—
Cough	2 (4.8%)	—	—	1 (2.4%)	—	—	> 0.999	—
Dyspnea	2 (4.8%)	—	—	—	—	—	0.494	—
Chest wall pain	—	—	—	1 (2.4%)	—	—	0.494	—
Pneumonitis	—	—	—	1 (2.4%)	—	—	0.494	—
**Skin**	9 (21%)	—	—	27 (66%)	5 (12%)	—	**< 0.001**	**0.026**
Atrophy	1 (2.4%)	—	—	3 (7.3%)	—	—	0.360	—
Dryness	1 (2.4%)	—	—	12 (29%)	1 (2.4%)	—	**< 0.001**	0.494
Edema	—	—	—	12 (29%)	3 (7.3%)	—	**< 0.001**	0.116
Erythema	2 (4.8%)	—	—	1 (2.4%)	1 (2.4%)	—	> 0.999	0.494
Hyperpigmentation	6 (14%)	—	—	24 (59%)	2 (4.9%)	—	**< 0.001**	0.241
Tenderness	2 (4.8%)	—	—	13 (32%)	—	—	**0.002**	—

Abbreviations: APBI = accelerated partial breast irradiation, WBI = whole breast irradiation

**Fig. 2:**
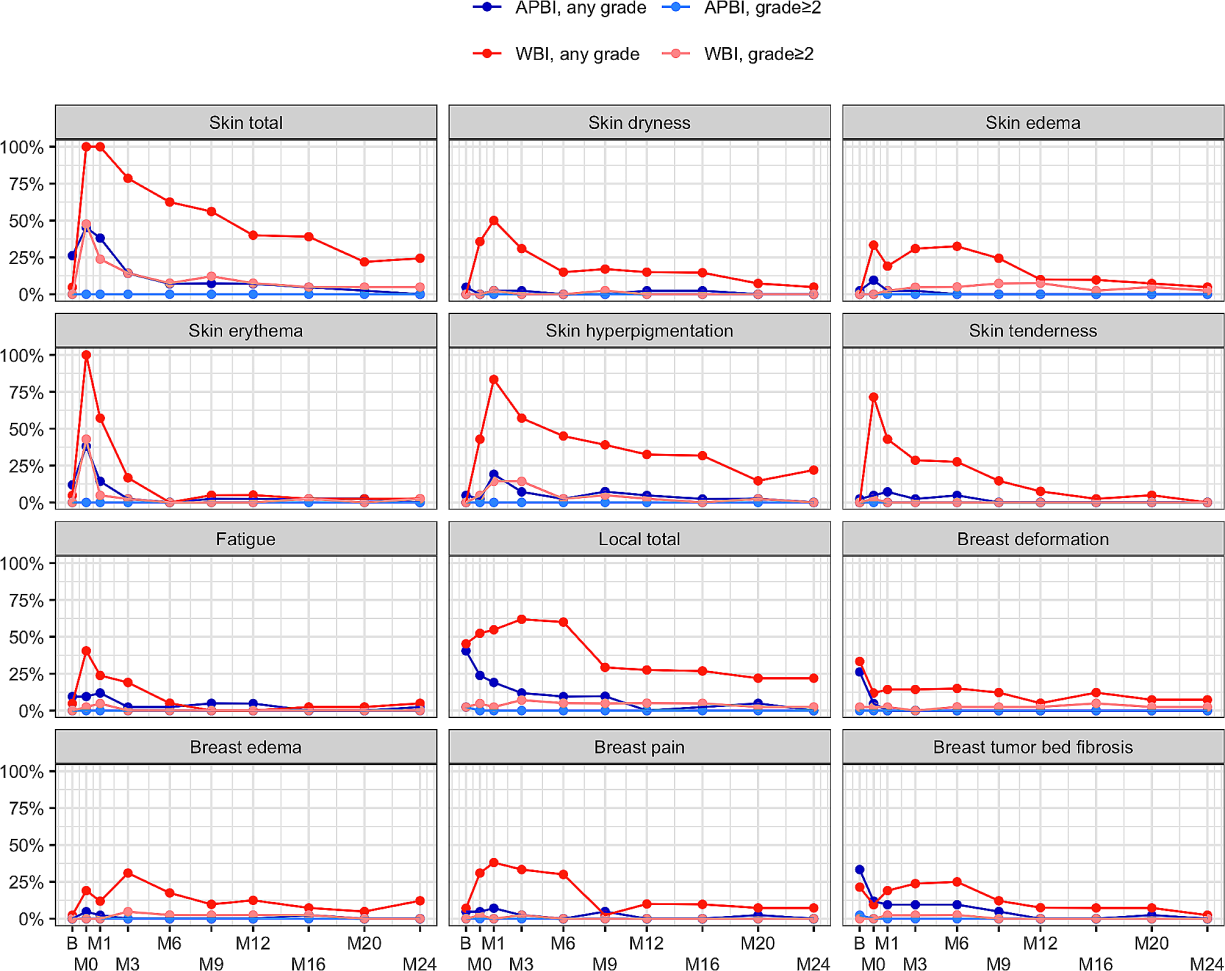
Time courses of toxicities by study arm and grade. Abbreviations: APBI = accelerated partial breast irradiation, WBI = whole breast irradiation

In the WBI arm, important late toxicity was worse and fading over time. A significant difference in favor of the study arm in skin dryness, edema, hyperpigmentation (*p* < 0.001 for all), and tenderness (*p* = 0.002) was observed. Grade ≥ 2 late skin toxicity developed in 5 (12%) patients in the WBI arm and none in the APBI arm (2-sided equality test: *p* = 0.026, noninferiority test with margin of 10%: *p* < 0.001). Late toxicity in the breast area (deformation, edema, fibrosis, and pain), affecting Qol and the cosmetic effect of the treatment, occurred in 61% of patients for any grade and 7.3% for grade ≥ 2 in the WBI arm and 17% for any grade in the APBI group (*p* < 0.001). Selected toxicities over time are shown in Fig. 2. More pronounced toxicity in the WBI arm is presented in all toxicity domains. Notably, grade ≥ 2 toxicities are minimal in the study arm. Fibrosis and breast deformation adverse events were common immediately after surgery and gradually disappeared over time, faster in the APBI arm, whereas in the WBI arm, reappeared after RT.

Based on CT scans six months after RT, radiographic signs of pneumonitis/fibrosis were less common in the APBI than in the WBI arm (1 patient, 2.4% vs. 26 patients, 63%, *p* < 0.001). One patient in WBI simultaneously had clinical symptoms of pneumonitis.

Cosmetic effects evaluated at different periods are summarized in Table 4. The cosmetic effect was more favorable in the APBI group, especially 6 to 12 months after the RT. A significant difference in the occurrence of cosmetic changes appeared between arms throughout the first year after irradiation. Major cosmetic changes (clear and severe differences) were noted mainly from the patient’s point of view (*p* = 0.003 at M0-M3 and *p* = 0.055 at M6-M12).

**Table 4:** Assessment of cosmetic effects by physician, nurse, and patient at different periods by study arm

	APBI				WBI				*p*-value	
	Nearly identical to untreated	Slightly different	Clearly different	Seriously distorted	Nearly identical to untreated	Slightly different	Clearly different	Seriously distorted	Any difference	Major difference*
**Baseline**										
Physician	33 (79%)	9 (21%)	—	—	37 (88%)	5 (12%)	—	—	0.380	—
Nurse	30 (71%)	12 (29%)	—	—	34 (81%)	8 (19%)	—	—	0.443	—
Patient	25 (60%)	14 (33%)	3 (7.1%)	—	26 (62%)	13 (31%)	3 (7.1%)	—	> 0.999	> 0.999
**M0-M3**										
Physician	21 (50%)	21 (50%)	—	—	9 (21%)	30 (71%)	3 (7.1%)	—	0.012	0.241
Nurse	20 (48%)	22 (52%)	—	—	10 (24%)	29 (69%)	3 (7.1%)	—	0.040	0.241
Patient	9 (21%)	32 (76%)	1 (2.4%)	—	7 (17%)	24 (57%)	10 (24%)	1 (2.4%)	0.782	0.003
**M6-M12**										
Physician	33 (79%)	9 (21%)	—	—	22 (52%)	18 (43%)	2 (4.8%)	—	0.021	0.494
Nurse	35 (83%)	7 (17%)	—	—	20 (48%)	20 (48%)	2 (4.8%)	—	0.001	0.494
Patient	26 (62%)	16 (38%)	—	—	9 (21%)	28 (67%)	4 (9.5%)	1 (2.4%)	< 0.001	0.055
**M16-M24**										
Physician	26 (62%)	16 (38%)	—	—	24 (59%)	17 (41%)	—	—	> 0.999	—
Nurse	26 (62%)	16 (38%)	—	—	23 (56%)	18 (44%)	—	—	0.824	—
Patient	20 (48%)	21 (50%)	1 (2.4%)	—	13 (32%)	26 (63%)	2 (4.9%)	—	0.266	> 0.999

*Comparing numbers of patients with clearly different and seriously distorted cosmetic effects

Abbreviations: APBI = accelerated partial breast irradiation, WBI = whole breast irradiation

## Discussion

This prospective randomized study of early BC patients is, together with the slightly earlier initiated HYPAB trial [33], the first to evaluate the efficacy and side effects of five-fractions ABPI (30 Gy in 5 fractions) compared to moderate hypofractionated, currently most used, WBI regimen (40 Gy in 15 fractions). The conclusions of our study and the HYBAB trial are consistent and help to increase the evidence for using this fractionation and technique in routine APBI practice. In contrast to the other trials using the same fractionation [6, 24], a dose of 30 Gy was administered in 5 consecutive daily fractions, VMAT technique and FFF beams were used for dose application.

Thanks to more precise irradiation using all available modern technologies and procedures used in targeted hypofractionated and stereotactic RT (surgical clips, CBCT, DIBH, VMAT, FFF, 6DoF couch), it was possible to reduce the volume of the PTV and thereby avoid the increased toxicity described in the oldest external APBI studies [13–15]. Therefore, the 3-mm PTV margin is supposed to be large enough to accommodate possible set-up errors in this setting. This is in line with the trend of modern radiotherapy, where safety margins can be reduced thanks to new technologies, thereby reducing side effects and improving the quality of life of oncology patients. Based on our findings, APBI is highly tolerable in regards to both toxicity and cosmetic effects, ultimately providing definite benefits to patients. As a result, the technique of external APBI may be used more often in clinical practice in the future. We are aware that confirmation of the oncological effectiveness of this technique and the appropriateness of using a 3-mm PTV margin requires a longer follow-up. However, the indication is that at the time of analysis, no local, regional, or distant recurrence of the disease was detected in both arms of patients.

Other randomized trials dealing with external APBI compared this technique with standard fractionated WBI (50 Gy in 25 fractions) [24, 34]. Comparing different fractionation schedules is also crucial. Studies involving twice-daily irradiation of patients reported increased toxicity and more adverse cosmetic effects [7, 8, 15]. Furthermore, our study demonstrated that consecutive irradiation yields favorable cosmetic outcomes with minimal toxicity, so irradiating every other day may not be necessary [35–37].

Adjuvant RT after primary surgery aims to eliminate the potential microscopic residual disease in the surgery bed and/or surrounding satellites [38, 39]. Recurrences occur most often at the site of the primary lesion [40, 41]. The randomized trials [6, 11, 24, 35, 42–47] have shown noninferiority in LC and OS after the tumor bed irradiation as opposed to WBI in patients with early BC after BCT [10–12, 48]. Based on these results, ASTRO and ESTRO recommend APBI as an alternative to WBI for selected patients with early BC [10–12, 49].

Published reputable studies [6, 46, 47] using external beam RT show better toxicity profiles and cosmetic effects in APBI arms. Livi et al. [24, 35, 42] compared the same APBI fractionation scheme (30 Gy in 5 fractions, every other day) with standard WBI (50 Gy in 25 fractions + boost). There was a significant difference in both any grade and grade ≥ 2 acute toxicity in favor of the APBI arm. The most frequently observed event was skin erythema (19.9% and 66.5% in APBI and WBI arms, respectively). Concerning late side effects, no grade ≥ 2 toxicity was observed in the APBI group. The most represented event was skin fibrosis in both arms (4.5% and 11.2% in APBI and WBI arms, respectively). The trial showed not only a significantly better toxicity profile but also the functional status and Qol after treatment and after 2 years were better in the APBI group, which is consistent with our study findings. We observed disappeared differences in cosmetic effects between WBI and APBI with longer follow-up. This may be due to the subjectivity of the assessment or the fact that patients have grown accustomed to the condition of their breast.

Some studies used 10 fractions in 5 days, i.e., twice daily irradiation [7, 8, 15], for external APBI. Data on patient preferences are limited, but both patients and physicians consider twice-daily radiation to be complicated and not optimal [50, 51]. The Canadian phase III RAPID study demonstrated the noninferiority of APBI in LC and acute toxicity. However, late toxicity and cosmetic results favored WBI. The authors concluded that the six-hour interval between fractions is too short for reparation. The APBI dose regimen used in our study (30 Gy in 5 fractions) is satisfactory for adjuvant irradiation. Qi et al. [52] described the α/β ratio (basic radiobiological parameter) of breast tumors to be relatively low (α/β = 2.88), and therefore high-dose RT can be very beneficial in the same way. Using a linear quadratic model and assuming an α/β ratio of 3 or 2.5, the prescribed dose used in our APBI study is equivalent to 54 or 56.7 Gy when using standard fractionation.

Since ASTRO and ESTRO recommendations were strictly followed [10–12, 48], only low-risk patients were included in APBI studies. Treatment results may be impaired if patients with a higher risk of recurrence (larger tumors, smaller surgical margins, hormone non-dependency, or lymph node involvement) are included. In the NSABP B-39/RTOG 0413 [8] study, enrolling patients with the nodal disease (pN1mic or pN1), criteria for noninferiority of the APBI were not reached in the number of ipsilateral recurrences (although the absolute difference was 0.7%).

The crucial factor affecting toxicity is the size of the target volume. Clinical trials using the 3D-CRT technique were associated with higher skin reactions and worse cosmetic results, particularly because of the need to accommodate extra safety margins to compensate for all inaccuracies during irradiation, including breast movements during breathing. The median PTV volumes in the published 3D-CRT studies were 269 cc, 296 cc, and 185 cc, respectively [53]. Livi et al. [24], as also mentioned above, used intensity modulated radiation therapy (IMRT) and showed no significant difference between PBI and WBI in ipsilateral breast tumor recurrence and survival rates at ten years, with significantly improved outcomes in treatment-related toxic effects and cosmetic results in favor of the APBI arm. The mean PTV volume in their trial was 139 cc (range 55–259). In our APBI trial, the VMAT technique was used for its accuracy and fast rate of dose application [19]. The median PTV volume in the APBI arm was 86.5 cc (range 40.9–189.9). The data presented from all the mentioned studies indicates that PTV volumes up to 150–180 cc are safe for applying the dose of 30 Gy in 5 fractions. Moreover, two studies used the same dose fractionation for WBI and PBI and showed that reducing the irradiated volume alone reduced late toxicity [6, 54]. 

A growing interest in ultra-hypofractionated regimens emerged during the COVID-19 pandemic [55]. A one-week hypofractionated WBI regimen has become standard in the UK. For local disease control, the WBI schedule of 26 Gy in 5 fractions over one week is non-inferior to 40 Gy in 15 fractions over three weeks [55–57]. Although it seems safe in terms of normal tissue effects for up to 5 years, this regimen has not been tested within a PBI phase 3 trial. In this context, the schedule of 30 Gy in 5 fractions represents an appealing treatment option that is both safe and effective [24, 34, 35, 37]. 

We are aware of the study’s limitations. The first is the relatively small number of patients, although sufficient for the statistical power of the considered objective. During the interim follow-up, there was no recurrence, regional or distant dissemination, or death of patients, but a longer follow-up for secondary objectives analysis is necessary. Second, the boost dose applied in our study is not necessary in light of current knowledge and recommendations and means certain overtreatment [58, 59]. When the study began, such a procedure was part of the treatment protocols at our institution. Also, most of the above-described studies used boost irradiation in the control arms. Recently, a boost may not be indicated in older patients with sufficient resection margins. This may have worsened the observed toxicity parameters in some patients and thus highlighted the differences between the arms. Our study was designed to confirm the non-inferiority of APBI compared to WBI. However, we observed that APBI patients experienced even significantly less toxicity.

Finally, several patients with a low-risk BC treated in the APBI trials may have been suitable candidates for the complete omission of adjuvant RT. Published meta-analyses established that forgoing WBI does not impact overall survival in selected patients but is associated with a significantly higher rate of LR [60–62]. This procedure is chosen mainly for patients with worse clinical conditions or comorbidities when a significant benefit from reducing the risk of ipsilateral disease recurrence using RT is not expected. On the other hand, the second pillar of early BC patient care is ET, which may negatively influence Qol of patients [63] due to its detrimental action on the cardiovascular system, bone density, sexuality, and cognition. Therefore, the oncological community is investigating [64] whether APBI could safely replace ET in very low-risk early BC (i.e., older age, luminal-A disease). In such a case, the double advantage of partial breast irradiation– a significant shortening of the total radiation time and less toxicity compared with WBI and the abolition of long-term toxicity of ET if omitted - would favorably affect QoL.

## Conclusion

The technique of external APBI using the principles of targeted hypofractional RT was found to be very well tolerated, easy to perform, and safe. External beam APBI schedule of 30 Gy in 5 fractions represents an attractive treatment option that is both safe and effective. Our study indicated that this technique may be a more feasible and less toxic option in the adjuvant setting for treating early BC patients compared to hypofractionated WBI, thus contributing to increasing its evidence for use in clinical practice. In the long term, short-course, once-daily external beam schedule will emerge as the favored approach to balance efficacy, convenience, and toxicity for those patients who proceed with adjuvant partial breast radiation.

### Electronic supplementary material

Below is the link to the electronic supplementary material.

**Supplement Material 1. Supplementary Table 1**: Dose constraints and plan optimization. **Supplementary Figure 1**: Dose distribution and beam arrangement for APBI arm

## Data Availability

No datasets were generated or analysed during the current study.

## Abbreviations

APBI: Accelerated partial breast irradiation
WBI: Whole breast irradiation
BC: Breast cancer
BCT: Breast-conserving therapy
PBI: Partial-breast irradiation
RT: Radiotherapy
LR: Local recurrence
BRT: Brachytherapy
3D-CRT: Three-dimensional conformal radiotherapy
IGRT: Image-guided radiotherapy
CBCT: Cone beam computed tomography
DIBH: Deep inspiration breath hold
VMAT: Volumetric modulated arc therapy
FFF: Flattening filter free beams
MMCI: Masaryk Memorial Cancer Institute
CTV: Clinical target volume
PTV: Planning target volume
CTCAE: Common Terminology Criteria for Adverse Events
Qol: Quality of life
CT: Computed tomography
IQR: Interquartile range
DCIS: Ductal Carcinoma In Situ
NST: Non-specified tumor histology
ER: Estrogen receptor
PR: Progesterone receptor
IA: Aromatase inhibitor
