# Supplementary material for: Toxicity of external beam accelerated partial-breast irradiation (APBI) in adjuvant therapy of early-stage breast cancer: prospective randomized study
Source: Radiat Oncol. 2024 Feb 3;19:17. doi: 10.1186/s13014-024-02412-x (PMC10837889; doi:10.1186/s13014-024-02412-x)
Supplement: Supplementary file 1 — Supplement Material 1. Supplementary Table 1: Dose constraints and plan optimization. Supplementary Figure 1: Dose distribution and beam arrangement for APBI arm [file 13014_2024_2412_MOESM1_ESM.docx]

**Supplementary material of Toxicity of External Beam Accelerated Partial-Breast Irradiation (APBI) in Adjuvant Therapy of Early-stage Breast Cancer: Randomized Prospective Study**

**Supplementary Table 1**: Dose constraints and plan optimization [1–4]

| **Dose constraints** | | | | | | | |
| --- | --- | --- | --- | --- | --- | --- | --- |
| **APBI arm** | | | | **WBI arm** | | | |
| **Organ** | Volume | Limit | Prescribe | **Organ** | Volume | Limit | Prescribe |
| **Ipsilateral breast** | 50%  35% | 15 Gy  30 Gy | V_15_ < 50%  V_30_ < 35% | **Ipsilateral breast** | NA | NA | NA |
| **Contralateral breast** | D_max_ | 1 Gy | D_max_ < 1 Gy | **Contralateral breast** | 10% | 5 Gy | V_5_ < 10% |
| **Ipsilateral lung** | 20 % | 10 Gy | V_10_ < 20% | **Ipsilateral lung** | 25%  10% | 20 Gy  30 Gy | V_20_ ≤ 25%  V_30_ ≤ 10% |
| **Contralateral lung** | 10 % | 5 Gy | V_5_ < 10% | **Heart** | D_mean_ | 2.5 Gy | D_mean_ < 2.5 Gy |
| **Heart**  **(right site)** | 5 %  10 % | 1.5 Gy  3 Gy | V_1.5_ < 5%  V_3_ < 10% | **Left ventricle** | D_mean_  17%  5 % | 3 Gy  5 Gy  23 Gy | D_mean_ < 3 Gy  V_5_ < 17%  V_23_ < 5% |
| **Heart**  **(left site)** | 40 %  10 % | 1.5 Gy  3 Gy | V_1.5_ < 40%  V_3_ < 10 % | **Left anterior descending artery** | D_mean_  2%  1% | 10 Gy  30 Gy  40 Gy | D_mean_ < 10 Gy  V_30_ < 2%  V_40_ < 1% |
| **Chest wall** | D_max_ | 36 Gy | D_max_ < 36 Gy |  |  |  |  |
| **Skin** | D_max_ | 36 Gy | D_max_ < 36 Gy |  |  |  |  |
| **Plan optimization** | | | | | | | |
| 100% of prescribed dose covers ≥ 95% of PTV  D_near-max_ to PTV < 105% (D_2%_ < 31.5 Gy)  D_near-min_ to PTV > 28 Gy (D_98%_ > 28 Gy) | | | | | | | |

Abbreviations: APBI = accelerated partial breast irradiation, WBI = whole breast irradiation, Gy = Gray, NA = not applicable, PTV = planning target volume, V_x_ = volume of appropriate dose, D_mean_ = mean dose of the volume, D_max_ = maximum dose of the volume, D_near-min_ = near-minimum dose of the volume, D_near-max_ = near maximum dose of the volume. D_near-min_ and D_near-max_ referred according to ICRU report 83 [5].

**Supplementary Figure 1:** Dose distribution and beam arrangement for APBI arm


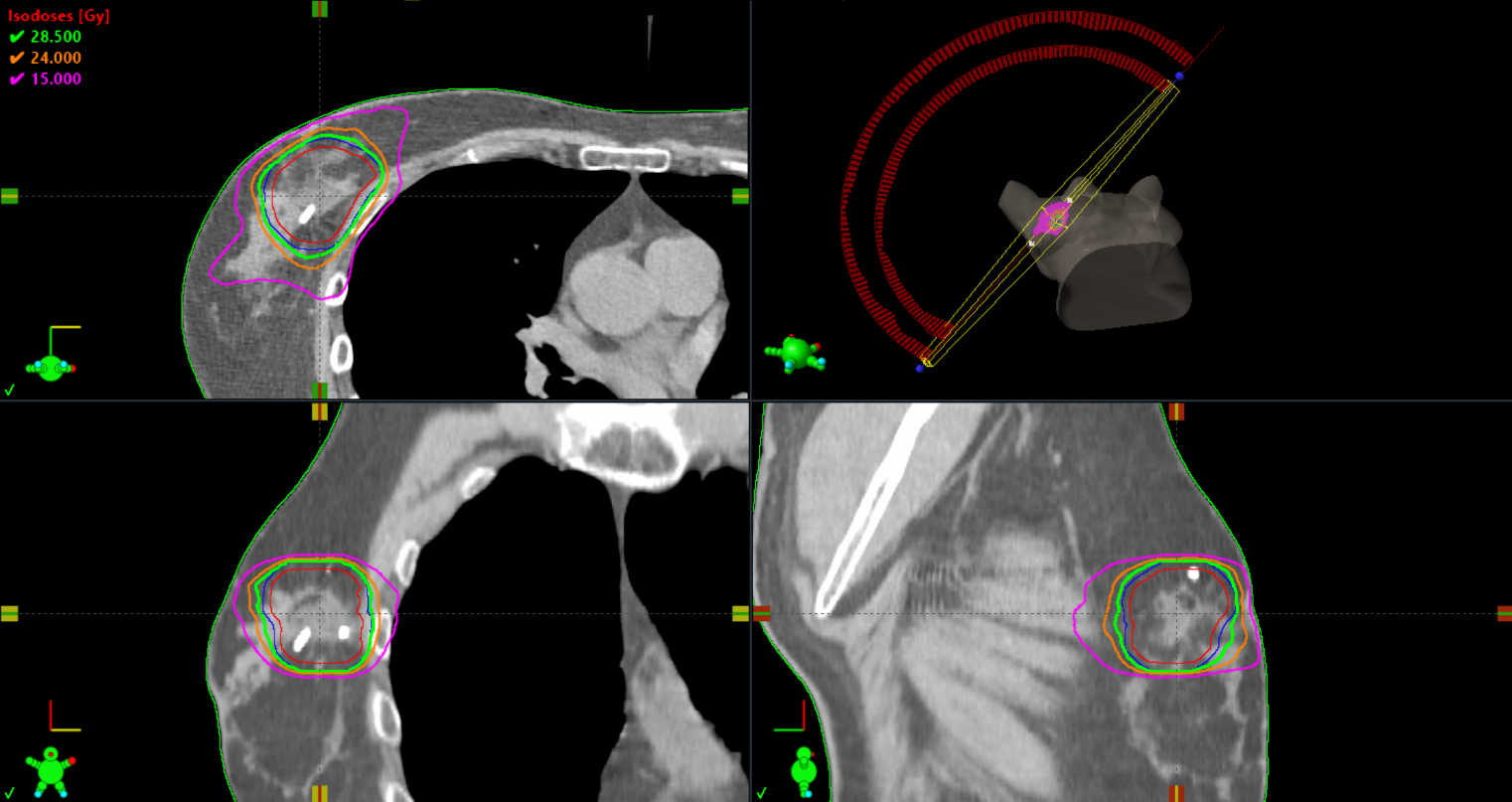


CTV (red line) = 41.7 cc, PTV (blue line) = 64.5 cc, D_min_ in CTV = 29.5 Gy, D_near-min_ in CTV = 30.1 Gy, Dmin in PTV = 28.2 Gy, D_near-min_ in PTV = 29.7 Gy, D_mean_ in PTV = 30.4 Gy, D_near-max_ = 31.2 Gy, D_max_ = 31.9 Gy, appropriate isodose lines (95% of prescribed dose = green, 80% of prescribed dose = orange and 50% of prescribed dose = violet line).

**References:**

[1] Livi L, Meattini I, Marrazzo L, Simontacchi G, Pallotta S, Saieva C, et al. Accelerated partial breast irradiation using intensity-modulated radiotherapy versus whole breast irradiation: 5-year survival analysis of a phase 3 randomised controlled trial. Eur J Cancer 2015;51:451–63. https://doi.org/10.1016/J.EJCA.2014.12.013.

[2] Obayomi-Davies O, Kole TP, Oppong B, Rudra S, Makariou E V., Campbell LD, et al. Stereotactic Accelerated Partial Breast Irradiation for Early-Stage Breast Cancer: Rationale, Feasibility, and Early Experience Using the CyberKnife Radiosurgery Delivery Platform. Front Oncol 2016;6:23. https://doi.org/10.3389/FONC.2016.00129.

[3] Piroth MD, Baumann R, Budach W, Dunst J, Feyer P, Fietkau R, et al. Heart toxicity from breast cancer radiotherapy : Current findings, assessment, and prevention. Strahlenther Onkol 2019;195. https://doi.org/10.1007/S00066-018-1378-Z.

[4] Lee BM, Chang AS, Kim SY, et al. Risk of radiation pneumonitis following individualized modern radiotherapy with IMRT, a breath-holding technique, and prone positioning for breast cancer. Abstract selected for 2018 Best of ASTRO (November 30-December 1, 2018) from ASTRO’s 60th Annual Meeting (October 21-24, 2018). n.d.

[5] Gregoire V, Mackie TR. State of the art on dose prescription, reporting and recording in Intensity-Modulated Radiation Therapy (ICRU report No. 83). Cancer/Radiothérapie (2011) 15:555–9. doi: 10.1016/j.canrad.2011.04.003
